# Retinal vessel density and cognitive function in healthy older adults

**DOI:** 10.1007/s00221-025-07076-x

**Published:** 2025-04-15

**Authors:** Dieter F. Kutz, Justus Obergassel, Melanie Mack, Robert Stojan, Boris Schmitz, Florian Alten, Claudia Voelcker-Rehage

**Affiliations:** 1 Department of Neuromotor and Exercise, University of Münster, 48149 Münster, Germany; 2 Otto Creutzfeldt Center for Cognitive and Behavioral Neuroscience, University of Münster, 48149 Münster, Germany; 3 Department of Ophthalmology, University of Muenster Medical Center, Muenster, Germany; 4 University of Geneva – Center for the Interdisciplinary Study of Gerontology and Vulnerabilities (CIGEV), Geneva, Switzerland; 5 Faculty of Health, Department of Rehabilitation Sciences, University of Witten, Herdecke, Witten, Germany; 6 DRV Clinic Königsfeld, Center for Medical Rehabilitation, Ennepetal, Germany; 7 JICE, Joint Institute for Individualisation in a Changing Environment, University of Münster and Bielefeld University, Münster, Germany

**Keywords:** Ageing, MMSE, MoCA, Optical coherence tomography

## Abstract

The eye is considered a gateway to the brain, as the retina is the only tissue of the central nervous system not protected by bones. This enables non-invasive imaging to provide exceptional insights into the brain. Based on the similarity of brain and retinal structure, it is being investigated whether changes in retinal blood flow could serve as a potential biomarker for cognitive decline. Optical coherence tomography angiography (OCTA) enables an examination of the microcirculation of the retina on the basis of retinal flow registration. Retinal blood flow has been associated with cognitive function and changes have been shown for Alzheimer’s patients, whereas data for healthy older subjects are contradictory. The aim of this study was to correlate OCTA parameters with the individual performance in tests assessing the executive functions (EFs) inhibition, updating and shifting in a group of healthy older adults (range: 65–79) using structural equation modeling, with hematocrit serving as a mediator. A model was obtained for vessel density (VD) of the retinal superficial vascular complex and the EF parameters inhibition and updating. The model revealed that only the mediator hematocrit correlated with EF, whereas neither the direct path VD to EF nor the indirect path VD via hematocrit to EF were significantly correlated. Regression analysis with hematocrit yielded significant results for the variable updating. We conclude that higher levels of hematocrit shorten reaction time and the coefficient of variation increases correspondingly, indicating a positive effect of hematocrit on EF.

**Supplementary Information:**

The online version contains supplementary material available at 10.1007/s00221-025-07076-x.

## Introduction

The eye is considered a gateway to the brain, as the neural components are ontogenetically viewed an outgrowth of the diencephalon, making the retina the only tissue of the central nervous system not protected by bones. This allows for non-invasive imaging that provides exceptional insights into the brain (Hussain et al. 2023). The retinal pigment epithelium forms the outer blood-retinal barrier, separating the retina from the choroid. The blood supply to these components differs. The choroid is supplied by the short posterior ciliary arteries, while the retina is supplied by the central retinal artery. The latter enters the eyeball at the optic disc along the optic nerve and supplies the inner retina. Imaging techniques of the retina have made rapid progress and enable non-invasive assessments of the retinal vessels and neuronal structure using optical coherence tomography (OCT) and optical coherence tomography angiography (OCTA). OCT is a high-resolution imaging technique producing two- or three-dimensional images i.e. of the retina and optic nerve similar to an ultrasound scan (Huang et al. 1991). OCTA enables examination of the microcirculation of the retina and choroid based on its flow registration (for review see Lang et al. 2016; Koustenis et al. 2017; Pircher and Zawadzki 2017; Leitgeb 2019) and thus the determination of the vessel density (VD) of the vascular plexuses and the size of the foveal avascular zone (Dong et al. 2022). The retinal microvasculature can be divided into a superficial vascular complex (SVC) consisting of the radial peripapillary capillary plexus and superficial vascular plexus (SVP), and the deep vascular complex (DVC) consisting of the intermediate and deep capillary plexus (Campbell et al. 2017). The VD of the vascular plexus decreases to varying degrees with increasing eccentricity from the fovea to the periphery (Lavia et al. 2020). The decrease in VD of the SVP corresponds to the decrease in ganglion cell density. The VD of the intermediate capillary plexus decreases progressively from the fovea and disappears at an eccentricity of 8 to 9 mm laterally. In contrast, the deep capillary plexus only shows a slightly reduced VD towards the retinal periphery (Lavia et al. 2020).

Considering the similarity and continuity in the structure of the brain and the retina, as well as the cerebral and retinal vessels, it has been investigated whether changes in the structure or blood flow of the retina could serve as potential biomarkers for changes in overall intracranial blood flow (Xu et al. 2023; Csiszar et al. 2024; Wang et al. 2024a, 2024b; Wei et al. 2024) and specifically for cognitive decline, as it occurs in Alzheimer’s disease (AD) (Ashimatey et al. 2020; Yan et al. 2021; Jeevakumar et al. 2022; Wang et al. 2022; Wisely et al. 2022, 2024; Hussain et al. 2023; Reboucas et al. 2023). In a recent systematic review (Jeevakumar et al. 2022), the association between retinal markers and cognitive impairment in AD was examined in 67 publications, 58 based on OCT and 18 on OCTA. A significant correlation between the imaging parameters and cognitive performance (CP) was found in 58 publications with OCT and 18 with OCTA. CP was determined in 58 of 67 publications using the Mini-mental state examination (MMSE). When specifically considering the correlation between VD and CP, only 7 out of 14 publications showed a significant result, with 6 of these 7 publications using the MMSE (Creavin et al. 2016) exclusively to classify the cognitive status of the participants (Jeevakumar et al. 2022). Only one study reported regression from MMSE to VD (Bulut et al. 2018). For mild cognitive impairment, only a few studies (Bulut et al. 2018; Chua et al. 2020; Biscetti et al. 2021) showed a correlation between a decrease in CP and VD. For CP in healthy older participants, contradictive findings have been reported. Fang et al (2021) showed a correlation between the Montreal Cognitive Assessment (MoCA) (Nasreddine et al. 2005) and the VD of the SVP. Wang et al (2022) came to a comparable conclusion only indirectly, as they found correlations between OCTA parameters and neuroimaging parameters as well as correlations between neuroimaging parameters and CP. In contrast, other studies suggest that correlations between CP and OCT parameter are essentially genetically determined (Jones-Odeh et al. 2016), and that OCTA parameters do not show any correlation with CP (Abraham et al. 2021).

The described heterogeneity among healthy participants may be caused by differences in the applied CP tests, since most tests measure global cognition (e.g. MMSE, MoCA) and not performance in certain subdimensions of cognition such as executive functions (EFs). Thus, the aim of this study was to relate OCTA parameters with individual performance on tests evaluating EF, specifically inhibition, updating, and shifting, instead of relying solely on global cognitive measures like the MMSE. Structural equation modeling (SEM) was used to investigate the extent to which VD can predict EF performance, while modeling the complexity of the direct and indirect relationships between variables of interest. VD is set as a latent variable of selected OCTA parameters and EF as latent variables by inhibition, updating and switching performances. Since it is known that the hematocrit level affects OCTA measurement (Yang et al. 2017; Nelis et al. 2019) and that an increased VD improves blood and oxygen supply, hematocrit was determined. An increased VD was assumed to have both a direct effect on EF and an indirect effect via the hematocrit. Hence, hematocrit was used as a mediator in the SEM. The resulting model (Model 1) was compared with a model in which the individual MMSE score as a measure of global cognition was used instead of the latent variable EF (Model 2) and a model combining the specific EFs and global cognition (Model 3).

## Methods

This study is part of a larger project at the University of Münster, Germany, focusing on cognitive-motor multitasking in older adults (Mack et al. 2022). This sub-study was performed between January 2022 and November 2022. Eligibility criteria and cognitive tests are described in detail elsewhere (Mack et al. 2022). In brief, interested participants were assessed for eligibility through a standardized telephone interview, querying the inclusion and exclusion criteria described below. All participants gave written informed consent before participating in the study and received financial compensation (€15 per test day). Inclusion criteria were: (1) age between 65 and 75 years (minor exceptions are made for couples for ethical reasons: < 65 and > 75 years, n = 1), (2) right-handedness and (3) willingness to participate in the OCTA study. Exclusion criteria were: (4) red-green deficiency or red-green color blindness, (5) perceived health concerns, (6) neurological disease, stroke and/or head/brain surgery. Additionally, participants were screened for visual acuity using the Freiburg Visual Acuity Test version 3.9.0 with a cut-off of 20/50 (Bach 2007). They were also asked about their medication use (Supplementary Table 1). Furthermore, participants were required to obtain medical clearance, including a stress electrocardiogram (ECG), from a physician or cardiologist to perform a cardiorespiratory fitness test (cycling spiroergometry). Blood pressure was monitored throughout the warm up, graded exercise testing and cool down phases (cycling spiroergometry).

### Tests of global cognition and executive functions

Global cognition was assessed using the 30-point scale of the Mini-Mental State Examination (Folstein et al. 1975) (30 points = no cognitive impairment). A score < 25 was set as the threshold for mild cognitive impairment (Creavin et al. 2016). The test of the executive functions inhibitory control (‘inhibition’), working memory updating (‘updating’), and shifting were described elsewhere (Mack et al. 2022) and are briefly repeated here. The tests were computerized and conducted on a 24″ screen with a display resolution of 1920 × 1080 pixel and a screen distance of about 65 cm. Each test took about 10 min with up to three practice trials of about 1 to 2 min each. Feedback was provided after practice trials, but not after registered trials. For the assessments, visual (shifting) or visuo-spatial stimuli (inhibition, updating) were used, programmed in E-Prime 2.0 (Psychology Software Tools, Pittsburgh, PA, USA). The stimuli were presented in six blocks with breaks of 5 s between blocks (20 s after block 3). The maximum response window was 2000 ms. After a response or after a maximum of 2000 ms, a central fixation cross (0.3 cm width and height) was presented for a variable response-stimulus interval between 800 and 1200 ms. Participants responded by pressing the “X” or “M” key on a German keyboard with their left or right index finger, respectively. They were instructed to respond as fast and as accurately as possible. Reaction times and correctness of responses were recorded.

*Inhibition* was assessed using a modified Simon test. A black fixation cross was presented continuously on a white screen. At the beginning of a trial (n = 32 per block, 192 in total) an arrow (2 cm length, 0.5 cm height) pointing to the left or right was displayed for 500 ms either on the left or right side of the fixation cross (distance between arrows and fixation cross was 3.1 cm). For one half of the stimuli the direction and position of the arrow was congruent (congruent trials; e.g., leftward pointing arrow on the left side), while for the other half of the stimuli, direction and position was incongruent (incongruent trials; e.g., leftward pointing arrow on the right side, n = 96). Participants were instructed to press the left key “X” for leftward pointing arrows and the right key “M” for rightward pointing arrows, regardless of the stimulus position relative to the fixation cross.

*Updating* was assessed using the 2-back condition of a visuo-spatial working memory N-back test. A black 4 × 4 grid (18.4 cm width and height) was presented continuously. Dots (n = 19 per block, 2.6 cm diameter, 114 in total) were presented sequentially in the center of different grid cells (4.6 cm width and height) for 500 ms. Participants were instructed to memorize the position of the dots and to press the right key “M” when the position of the current dot was identical to the position of the second-to-last dot (target). They had to press the left key “X” when the current dot appeared at a different position as the second-to-last dot (non-target). In total, 30 targets and 72 non-targets were presented (plus 2 “starting trials” per block).

*Shifting* was assessed using a modified visual switching test. Geometrical shapes (n = 17 per block, 102 in total) were presented sequentially for 1500 ms in the center of the screen. The geometrical shapes were either quadratic or circular and either large (5.2 cm diameter/width and height) or small (1.8 cm diameter/width and height). Participants were instructed to indicate either the size of the shape (subtask A) or the form of the shape (subtask B) by pressing either the left key “X” for small or circular shapes or the right key “M” for large or quadratic shapes. In each block subtasks were presented in the following order: AABBAABBAABBAABBA (AA, BB: repetition trials; AB, BA: switch trials). No external cues about subtask order were provided.

### OCTA measures

OCTA imaging was performed with the AngioVue OCT and OCTA imaging system (RTVue-XR Avanti optical coherence tomograph, Optovue Inc., Fremont, CA, USA). Only scans with an overall scan quality ≥ 5 and signal-strength-index > 50 were accepted. An active eye-tracking technology was used to reduce motion artefacts (Lauermann et al. 2017). Standardized, the right eye was measured unless ophthalmic conditions (i.e. cataract) reduced insight and aforementioned quality indices could only be achieved by measuring the left eye (n = 6 participants). For angiographic imaging of the macula, 3 mm × 3 mm scans, for imaging of the optic disc, 4.5 mm × 4.5 mm scans were obtained, respectively, and the retinal layers were segmented automatically using the integrated software (Lauermann et al. 2018). The custom boundaries for the superficial vascular complex (SVC) were set between the inner limiting membrane (ILM) and 9 µm above the inner plexiform layer (IPL), for the deep vascular complex (DVC) between 9 µm above the IPL and 9 µm below the outer plexiform layer and for the radial peripapillary capillaries (RPC) between ILM and below nerve fiber layer. The peripapillary region is defined as an annulus with an outer diameter of 4 mm and inner diameter of 2 mm around the optic disc, the parafoveal region as an annulus with an outer diameter of 3 mm and an inner diameter of 1 mm. The en-face images of the optic disc, as well as the OCT images of the macula revealed no pathological abnormalities of the optic nerve or macula. The intraocular pressure was not measured prior to the OCTA assessments – however, patients were questioned regarding previous ophthalmological diagnoses to exclude glaucoma and other relevant ocular pathologies.

Since hematocrit level has been reported to affect OCTA measurement, venous blood samples were drawn (K3-EDTA) with a time interval of no more than 7 days from OCTA analyses. Analysis was performed in the accredited laboratory of the University Hospital Münster, Germany, using standard procedures.

Various OCTA parameters can be extracted from the software as mentioned elsewhere (Zinn et al. 2020; Alten et al. 2021). For this study, OCTA parameters were selected according to the principle that the VD in the examined area should be as uniform and undisturbed as possible in order to have a comparable situation to the microcirculation in the brain. Therefore, the areas of the papilla (optic disc) and the fovea were excluded. The selected areas were the parafoveal area between a 1 mm and 3 mm diameter around the fovea with differentiation between SVC and DVC as well as the peripapillary capillaries with recess of the optic nerve head.

### Data analysis

For all trials with correct responses, a measurement of speed (RT of correct responses), accuracy (percentage of correct responses) and variability (coefficient of variation, CV across reaction times of correct trials) was extracted for each executive function tested. The selected parameters and their definitions are given in Table 1. For each test, outliers of individual performance were eliminated before the results were calculated by first removing trials with reaction times < 80 ms or > 1300 ms for the individual participant and then using a two-sided 1 ‰ criterion to exclude values that were more than 3.29 times the standard deviation from the mean value of the participant. Accuracy was quantified as the percentage of correct responses across all presented stimuli and speed as the mean reaction time of correct responses. CV, defined as the ratio of the standard deviation (SD) to the mean, was calculated by dividing the SD of RTs by the average RT of the participant. To verify participants’ understanding of the tasks, we examined whether the mean accuracy across all trials (Simon task: congruent and incongruent trials, n-back: target and non-target trials; visual switching: repetition trials and switch trials) for participants exceeded 55%. Next, we calculate cost values by subtracting the RT/CV/accuracy of the incongruent trials from the congruent trials (Simon task), the RT/CV/accuracy of the non-target trials from the target trials (n-back task), and the RT/CV/accuracy of the switch trials from the non-switch trials.

**Table 1:** Selected parameters of the cognitive tests (Simon inhibition task, n-back working memory (WM) task, and visual switching task) to assess the executive functions inhibition, updating and shifting

Executive function	Variable	Definition
Inhibition	Inhibition-costs_speed	Mean RT of correct congruent trials –Mean RT of correct incongruent trials
	Inhibition-costs_variability	CV of RT of congruent trials –CV of RT of incongruent trials
	Inhibition-costs_accuracy*	Percentage of correct congruent trials –Percentage of correct incongruent trials
Updating	Updating_speed	(Mean RT of correct target trials + Mean RT of correct nontarget trials)/2
	Updating_variability	CV of mean RT of all correct target and nontarget trials
	Updating_accuracy	(Percentage of correct target trials + Percentage of correct nontarget trials)/2
Shifting	Switch-costs_speed	Mean RT of correct repetition trials –Mean RT of correct switch trials
	Switch-costs_variability	CV of RT of repetition trials –CV of RT of switch trials
	Switch-costs_accuracy	Percentage of correct repetition trials –Percentage of correct switch trials

*CV* coefficient of variation, *RT* reaction time, *WM* working memory. *Parameter was transformed logarithmically

All statistical analysis were performed using the R 4.3.2 base package (Team 2018, 2024). For mediation analysis, structural equation modeling (SEM) was done using the R-package *lavaan* 0.6–15 (Rosseel 2012). To meet minimum requirements for an SEM, the respective parameters either had to be approximately normally distributed or were adjusted accordingly through suitable transformation (e.g., logarithmic transformation). Subsequently, individual values that deviated by more than 2.17 SDs from the group mean were identified as outliers and excluded from the SEM using a two-sided 1.5% criterion. The three selected OCTA parameters were used as parameters for the latent variable VD. It is possible that elevated blood pressure may have affected the OCTA measurement. In addition, some participants were taking antihypertensive medications (see Supplement Table 1 for list of medications). Therefore, we selected systolic blood pressure (RRsys) measured under similar conditions in all participants as a covariate for vessel density (VD). We chose the minimum RRsys value recorded during the warm-up period at 0W power output because, based on previous experience, participants tend to have their lowest values during this period. Earlier blood pressure readings tend to be high due to participants’ tension about the unfamiliar laboratory environment. For the first model (Model 1), the latent variable EF was formed hierarchically with two levels. The top level was formed by the executive functions inhibition, updating, and shifting as latent variables. On the second level, each individuals’ executive function formed its own latent variable, which was based on three selected parameters (Table 1). Age and years of education were included as covariates in the model and hematocrit level as a mediator of the relationship between VD and executive functions. As alternative models, SEMs were examined by replacing the latent variable EF with the MMSE score (Model 2), as well as a model in which the latent variable cognition consisted of the MMSE score combined with the latent variables inhibition, updating, and shifting. Model optimization and evaluation followed standard procedures of confirmatory factor analysis (Schermelleh-Engel et al. 2003; Gäde et al. 2020). The occurrence of negative variance estimates (so-called Heywood cases) was considered a convergence problem or an incorrect solution of the model. In these cases, further parameters were eliminated until a stable model was determined (Schermelleh-Engel et al. 2003). For comparing the models robust estimates of the χ^2^ value, the Comparative Fit Index (CFI), and the Tucker-Lewis Index (TLI), as well as the sample-size adjusted Bayesian Information Criterion (SABIC) were used (Schermelleh-Engel et al. 2003; Gäde et al. 2020).

## Results

Forty-one participants took part in this sub-study. Due to missing data (n = 2) or exclusion due to outliers (n = 5), only data from 34 participants were included in the analysis. The demographic data of participants included in this study are shown in Table 2.

**Table 2:** Demographic data (N = 34, 21 females, 13 males)

	Mean ± sd^1)^	Median / iqr^2)^, range
Age (years)	69.6 ± 3.4	69/6, [65, 79]
Education (years)	16.2 ± 3.7	17/4, [11, 25]
MMSE	28.7 ± 1.3	29/2, [26, 30]
Hematocrit (%)	42.5 ± 3.9	41.6/5.1, [36.1, 51.6]

^1)^Standard deviation;, ^2)^Inter-quartile range

**Fig. 1:**
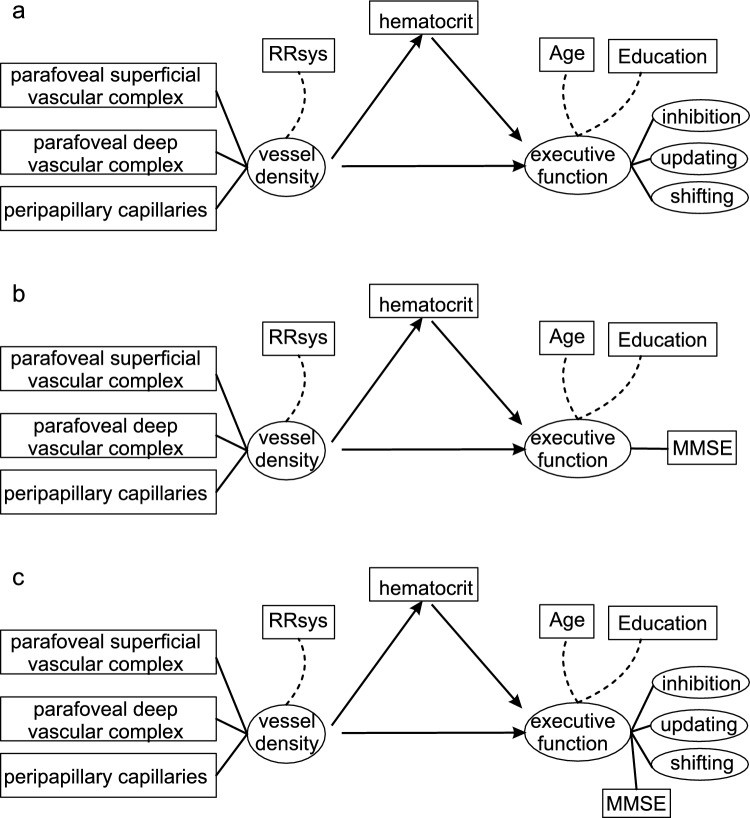
Proposed structural equation models **a** Model 1: latent variable executive function formed from inhibition, updating and switching, **b** Model 2: latent variable executive function represented by the global cognition score MMSE, **c** Model 3: combination of models 1 and 2

**Fig. 2:**
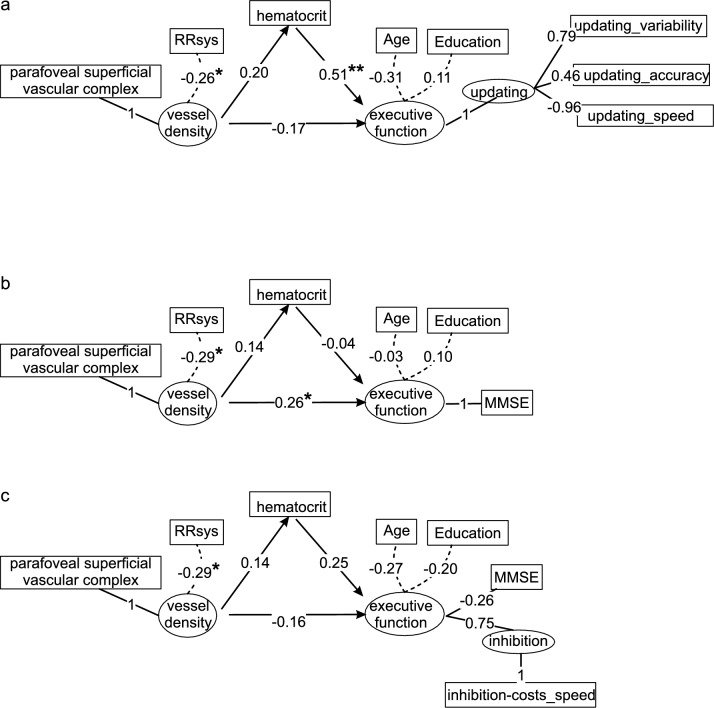
Results of the structural equation models **a** Model 1, **b** Model 2, **c** Model 3. The figures show the standardized weights across all levels. significant code: ‘*’p < 0.05, ‘**’p < 0.01

SEMs were conducted to investigate the extent to which retinal VD predicts executive function performance, using the hematocrit level as a mediator. The latent variables of VD were the VD of the parafoveal superficial and deep vascular complexes as well as the peripapillary capillaries. As some of the participants were taking antihypertensive medication (N = 10), RRsys was used as a covariate for the VD measurements. For the first model, the latent variable EF was formed from inhibition, updating and shifting (Fig. 1a) with age and education as covariates. The SEM revealed that the VD of the parafoveal DVC and of the peripapillary capillaries did not load on the latent variable VD. Furthermore, it was observed that the executive function “shifting” did not load onto latent variable EF. Furthermore, latent variable inhibition was excluded because it was a source of negative variance in the executive function variable. Therefore, these variables were eliminated from all models. The model fit of the reduced model 1 (Fig. 2a) was assessed as good with χ^2^/degree of freedom = 19.635/19 = 1.03 (Schermelleh-Engel et al. 2003; Gäde et al. 2020). The analysis of the reduced model indicated that VD neither regressed directly (p = 0.247) nor indirectly (p = 0.361) with EF (see Supplement File 2). Hence, no total effect was detected. In contrast, the regression of hematocrit on EF was significant (p = 0.005). VD was also found to significantly covary with RRsys (p = 0.027).

As alternative models, a SEM was constructed in which the latent variable EF was represented by the MMSE score (Model 2). The proposed model is shown in Fig. 1b and the final reduced model in Fig. 2b. The model fit of the reduced model 2 (Fig. 2b) was assessed also as good with χ^2^/degree of freedom = 8.612/9 = 0.96. In this case, only the direct path (VD—> MMSE) was significant (p = 0.049, Fig. 2b). Model 3, the combination of the two models (proposed model: Fig. 1c, final reduced model: Fig. 2c) only provided a suitable approach after excluding the variable updating. Only the indicator inhibition-costs_speed was used for the latent variable inhibition, as the other two indicators resulted in negative variances of the variable executive function. The goodness of fit of the reduced model 3 is also satisfactory, with a chi-square to degrees of freedom ratio of 0.83 (χ^2^/df). This model contained no significant regressions, neither in the direct path nor in the indirect path. Covariation between VD and RRsys was also significant in models 2 and 3 (p < 0.05, Supplement File 2).

The comparison of the ratio χ^2^/DoF as the sole measure for the evaluation of quality would lead to a ranking of the reduced models with the order 3, 2, 1. (Table 3). Including the robust estimates of the CFI and TLI indices in the evaluation revealed that Models 1 and 2 exhibited a favorable model fit (CFI ≥ 0.95, TLI ≥ 0.95, Schermelleh-Engel et al. 2003; Gäde et al. 2020)). According to the indices, Model 3, which does not include a significant regression, would also have to be considered a good fit (Schermelleh-Engel et al. 2003; Gäde et al. 2020). However, a robust TLI with a value of 6.159 indicates a mis-formulated model. Therefore, it is not considered further. The sample-size adjusted Bayesian Information Criterion (SABIC) exhibited a higher value for Model 1 than for Model 2 (Table 3). These differences were attributed to the varying number of degrees of freedom (DoF). When the DoF were taken into account, the Model 1 was identified as the most parsimonious. In addition, Model 1 demonstrated a variance explanation of 0.252, while Model 2 exhibited a variance explanation of 0.065 (Wolf et al. 2013). The complete printout of all model parameters is provided in Supplement File 2.

**Table 3:** Parameters for the quality of model fit

Modell	χ^2^	DoF	CFI	TLI	SABIC
1	19.635	19	0.986	0.979	2025.811
2	8.612	9	1.000	1.544	1284.149
3	10.858	13	1.000	6.159	1658.495

*DoF* degree of freedom, *CFI* Robust Comparative Fit Index, *TLI* Robust Tucker-Lewis Index, *SABIC* Sample-size adjusted Bayesian Information Criterion

**Fig. 3:**
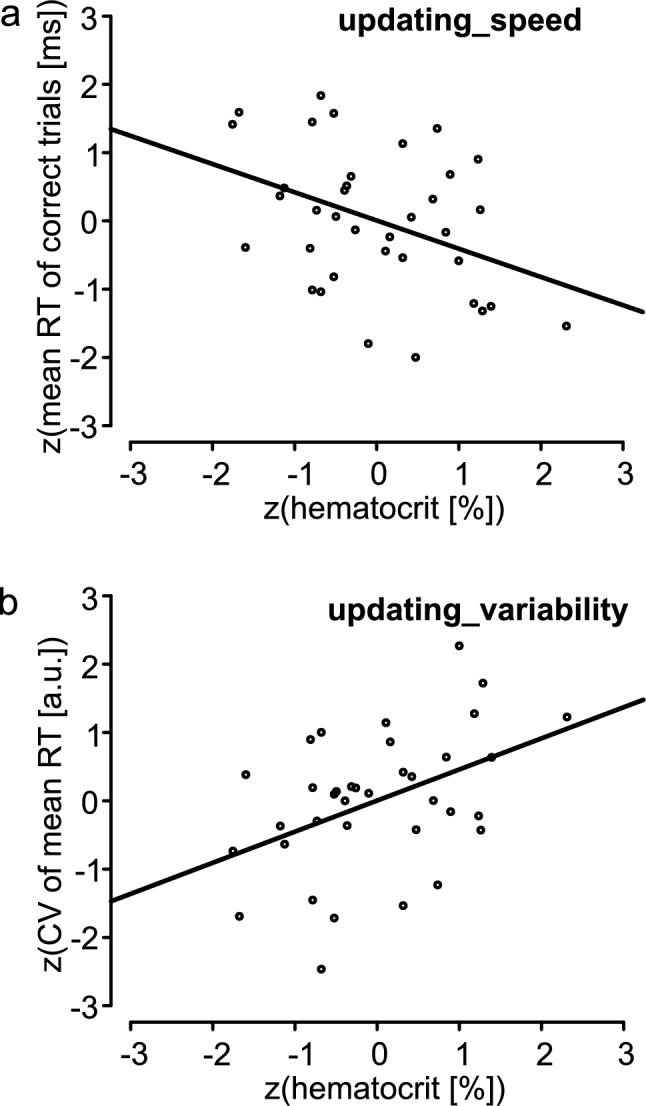
Regressions of hematocrit level with individual cognitive measurements. The regression was calculated on z-transformed data. For definitions of variables see Table 1

For Model 1, SEM results indicated that individual cognitive measures were at least indirectly dependent on hematocrit. To further investigate these potential dependencies, post-hoc regression analyses were conducted. The significant results are given in Table 4. For hematocrit, there were significant results for the Nback task with speed (Table 4: updating_speed) and variability (Table 4: updating_variability). This implied for the Nback task that faster responses occurred with higher hematocrit levels (Fig. 3a). Additionally, it is notable that the coefficient of variation (CV) demonstrated an increase with rising hematocrit levels (Fig. 3b).

**Table 4:** Results for the regression equations with different cognitive measures as dependent variable: variable ~ hematocrit

		Updating_speed	Updating_variability
	Slope	– 0.414 ± 0.165*	0.455 ± 0.161**
Goodness of fit	R^2^_adj_	0.13	0.17
	F(DoFs)	6.259 (1,34)	8.038 (1,34)
	*p*	0.017	0.008
	η^2^	0.16	0.19

The regression was calculated on z-transformed data. See Table 1 for definitions of variables.

Significant codes: ‘**’p < 0.01, ‘*’p < 0.05, R^2^_adj_ adjusted R^2^ value. *DoFs* degrees of freedom, η^2^ partial eta-square

## Discussion

This study investigated whether the individual performance of the EFs inhibition, updating, and shifting regresses with retinal vessel density (VD) assessed by high-sensitivity OCTA. Structural equation models were used to analyze the relationship between VD and EF, with hematocrit as a mediator. (Modell 1, Fig. 1a). The result of this model was compared with a model in which the individual MMSE score as a measure of global cognition was used instead of the latent variable EF (Model 2, Fig. 1b) and a model combining the specific EFs and global cognition (Model 3, Fig. 1c). For model 1, a viable model (Model 1) was obtained in which VD was described by the superficial vascular complex in the parafoveal retinal area and the EF parameters inhibition and updating (Fig. 2a). However, only the mediator hematocrit regressed significantly with EF. Neither the direct path VD on EF nor the indirect path VD via hematocrit on EF were significant. Thus, this study confirmed that it is not possible to infer EF from VD in healthy older subjects (e.g., Abraham et al. 2021). Of note, we identified a significant dependence of EF performance on hematocrit levels and it became evident that in the same proportion that mean reaction time decreases with increasing hematocrit (slope =  – 0.414 ± 0.165, Table 4, updating_speed), the CV increases (slope = 0.455 ± 0.161, Table 4, updating_variability). This suggests that the variation of reaction times remains constant. An improvement in oxygen supply can be conceptualised as supportive to all cells in the brain which in turn facilitates faster processing within the underlying network and ultimately resulting in shorter reaction times. Thus, it was shown that an increase in maximum oxygen uptake was associated with an improvement in cognitive performance. Concurrently, this was accompanied by a reduction in cortical activity in the superior temporal gyrus, the superior and medial frontal gyrus, and the anterior cingulate cortex (Voelcker-Rehage et al. 2011). However, improving the CV and thus inherently the individual variability requires improved connectivity within the network. Increasing hematocrit alone does not appear to be a sufficient means of achieving this. Overall, this indicates a positive effect of hematocrit levels on cognition. This dependence has only been shown in a few studies to date (Rafnsson et al. 2010; Ward et al. 2020). The hematocrit is a measure of oxygen transport capacity and thus a measure of the oxygen supply to the brain. It is noteworthy that the oxygen extraction fraction increases non-linearly with the hematocrit (Angleys and Østergaard 2020). It can therefore be assumed that the oxygen supply to cortical areas is a relevant parameter for EF performance.

It is noteworthy that including MMSE scores as a score of global cognition in the SEM (Model 2) led to a significant regression of the direct path (VD—> MMSE). Fang and colleagues (2021) only found a correlation between VD and the MoCA score, also a score of global cognition, in a similar group as in our study (N = 20, mean age = 71 years, range = [63–83]) while the correlation with MMSE was not confirmed. The authors argued this finding by stating that only the MoCA score had a sufficient large standard deviation, while the standard deviation of the MMSE score was too low, which led to the rejection of this correlation (Fang et al. 2021). Comparing the MMSE scores of the participants of this study with the participants of Fang et al (2021), the scores in our study were on average slightly lower (1 point) and more scattered. This was characterized by a higher coefficient of variation, which was about 4 times higher than in Fang’s study (0.0045 and 0.010, respectively).

The combination of both models was possible, whereby it was again shown that only the part hematocrit—> EF of the indirect path formed a regression at trend level. Incorporating the MMSE into the latent variable EF led to the exclusion of the execution function updating (Fig. 2c). Overall, the contribution of the MMSE to the latent variable EF was low with a relative value of  – 0.01. It should be noted that the MMSE examines different domains of cognitive performance in a generalized manner, with questions on declarative memory predominating. The chosen version of the Simon task in this study was specifically oriented towards procedural memory as the responses depended on the position of the stimulus. Therefore, the results found here are limited to procedural memory.

Critically, it should be noted that only the inhibition and updating tasks could be used to create a valid SEM, while shifting had to be excluded. It is important to consider that the tasks differ in their requirements. In the switching task, the design parameters shape and size of the stimuli were relevant for a correct response. Shape perception is located in the temporal cortex (Milner and Goodale 2006; Ayzenberg and Behrmann 2022a) whereas visual positioning and short-term memorization tasks are performed in the parietal cortex (Milner and Goodale 2006; Ayzenberg and Behrmann 2022b). Hence, it can be assumed that the different requirements of EF tasks engage these cortical areas differently. Importantly, arterial density in the temporal cortex is higher than in the parietal cortex (Bernier et al. 2018). Therefore, it can be assumed that the temporal cortex regulates the oxygen supply better during cognitive load and consequently is less dependent-and/or more flexible-on a sufficiently high hematocrit level. A further limitation of this study is evident for the co-variable education, for which no significant covariation to EF was found. It is noteworthy that the study participants were recruited via homepage announcements, by personal contact, senior college, local sports clubs as well as by advertisements in local newspapers, radio stations, and flyers (Mack et al. 2022). They therefore represent a biased selection of the average population in Germany. According to the Federal Statistical Office, for the distribution of educational qualifications by age group for the 65–75 age group, 27% had no formal education, 50% had completed school plus a vocational education and training, and around 23% had a higher education (Statistisches Bundesamt 2023). For the first group, the duration of education can be assumed to be less than 8 years, for the second group it corresponds to a training period of about 11–12 years and for the third group a duration of at least 12 years is assumed. The median duration of education of the participants in this study was 17 years and the minimum was 11 years, which indicates a significantly higher level of education. Therefore, the fact that hematocrit regresses with EF performance should be viewed with caution. This may be an effect of a selection bias of the study group and may not be applicable to all age groups.

A limitation of the analysis is the number of participants included. This meant that some indicators of latent variables had to be excluded, as otherwise this would have led negative variances in the overall result. Therefore, it cannot be ruled out that there is an effect of VD on latent variable switching, just as inhibition could still be relevant in model 1. Additionally, with a larger sample size, a meaningful model with significant regressions may also emerge for model 3. Nonetheless, significant regressions were found in model 1 (EF ~ hematocrit) and model 2 (EF ~ VD). Since the regression in model 1 with p < 0.01 is far from the classical limit of p < 0.05, a change in the direction of non-significance with an increase in the sample size would be highly unlikely at the moment. This supports our main conclusion that hematocrit has a positive effect on EF.

## Conclusion

It has been demonstrated that the vessel density of the OCTA is associated with global cognition measures (e.g. MMSE), yet not with the test performance on specific executive functions such as inhibition or updating. In specific tests, individual brain regions are activated. However, as a general measure, the OCTA does not provide information about the vessel density of these areas. Different factors, such as oxygen supply, play a crucial role for neuronal processing in these areas and therefore in performance in the specific tests. The hematocrit is a factor that also determines the oxygen supply to the brain. Therefore, the lack of association between VD and specific EFs suggests that changes in retinal VD are not a suitable parameter for predicting cognitive decline in healthy older adults. The observed changes of retinal vessel density found in AD (e.g., Jeevakumar et al. 2022) are to be regarded as specific findings of Alzheimer’s disease, and our results do not restrict the potential use of OCTA for the diagnosis of AD.

The task-dependent formation of a valid SEM supports the knowledge that different tasks are processed in distinct brain regions. It can be assumed that impairments in local cerebral oxygen supply lead to task-specific performance differences.

## Supplementary Information

Below is the link to the electronic supplementary material.

Supplementary file1 (DOCX 13 KB)

Supplementary file2 (PDF 76 KB)

## Data Availability

The data used in this study can be inquired from the corresponding author upon reasonable request.

## Abbreviations

CFI: Comparative Fit Index
CP: Cognitive performance
CV: Coefficient of variation
DoF: Degree of freedom
EF: Executive function
MMSE: Mini-mental state examination
MoCA: Montreal cognitive assessment
OCT: Optical coherence tomography
OCTA: Optical coherence tomography angiography
RT: Reaction time
SABIC: Sample-size adjusted Bayesian information criterion
SEM: Structural equation modeling
TLI: Tucker-Lewis Index
VD: Vessel density
