# Supplementary material for: Retinal vessel density and cognitive function in healthy older adults
Source: Exp Brain Res. 2025 Apr 15;243(5):114. doi: 10.1007/s00221-025-07076-x (PMC12000121; doi:10.1007/s00221-025-07076-x)
Supplement: Supplementary file 1 — Supplementary file1 (DOCX 13 KB) [file 221_2025_7076_MOESM1_ESM.docx]

Supplement table 1: List of medications taken. The use was coded binary (0=no, 1=yes).

medications for back injury (last 6 months)

medications for hand/arm injury (last 6 months)

medications for leg/foot injury (last 6 months)

medications for muscle skeletal system (last 6 months)

medications for rheumatic complaints (last 6 months)

medications for cardiovascular diseases (hypertension, coronary heart disease, heart attack, last 6 months)

medications for other cardiovascular diseases (last 6 months)

medications for neurological diseases (last 6 months)

medications for respiratory diseases (last 6 months)

medications for obesity (last 6 months)

medications for diabetes (last 6 months)

medications for increased blood cholesterol levels (last 6 months)

medications for other hormone and metabolic diseases (last 6 months)

medications for other disease (last 6 months)

postmenopausal estrogen therapy

regular intake of cholesterol/blood fat reducing medication

regular intake of tranquilizers

regular intake of mood elevators

regular intake of medications for sleep disorders

regular intake of antihypertensive medication

regular intake of other medication

name of drug

no regular intake of any medication
