# Supplementary material for: Retinal vessel density and cognitive function in healthy older adults
Source: Exp Brain Res. 2025 Apr 15;243(5):114. doi: 10.1007/s00221-025-07076-x (PMC12000121; doi:10.1007/s00221-025-07076-x)
Supplement: Supplementary file 2 — Supplementary file2 (PDF 76 KB) [file 221_2025_7076_MOESM2_ESM.pdf]

```
# Model 1
Mediation <- '# Mediation approach for executive_function ~ vascular density with haematocrit as mediator
              vascular_density =~ L1_ParaFovea_pre
        # executive_function as latent variable
             executive_function =~ updating
             updating =~ Nback.RT_CV + nback.ACC_wm + Nback.meanRT_wm
        # co-variances
             executive_function ~~ Age
             executive_function ~~ Education
             vascular_density ~~ RRsys
        # direct effect
              executive_function ~ c*vascular_density
        # haematocrit as Mediator
             HK_percent_pre ~ a*vascular_density
             executive_function ~ b*HK_percent_pre
        # indirect effect (a*b)
             ab := a*b
        # total effect
              total := c + (a*b)';

# call
model_fit <- sem(Mediation, data=mydata, estimator="mlm")
summary(model_fit, fit.measures = TRUE, standardized=TRUE)

lavaan 0.6-19 ended normally after 79 iterations

  Estimator                                         ML
  Optimization method                           NLMINB
  Number of model parameters                        17

                                                  Used       Total
  Number of observations                            36          41

Model Test User Model:
                                              Standard      Scaled
  Test Statistic                                20.293      19.635
  Degrees of freedom                                19          19
  P-value (Chi-square)                           0.377       0.417
  Scaling correction factor                                  1.034
    Satorra-Bentler correction

Model Test Baseline Model:

  Test statistic                                74.136      72.698
  Degrees of freedom                                28          28
  P-value                                        0.000       0.000
  Scaling correction factor                                  1.020

User Model versus Baseline Model:

  Comparative Fit Index (CFI)                    0.972       0.986
  Tucker-Lewis Index (TLI)                       0.959       0.979

  Robust Comparative Fit Index (CFI)                         0.986
  Robust Tucker-Lewis Index (TLI)                            0.979

Loglikelihood and Information Criteria:

  Loglikelihood user model (H0)              -1008.999   -1008.999
  Loglikelihood unrestricted model (H1)       -998.853    -998.853

  Akaike (AIC)                                2051.999    2051.999
  Bayesian (BIC)                              2078.919    2078.919
  Sample-size adjusted Bayesian (SABIC)       2025.811    2025.811

Root Mean Square Error of Approximation:

  RMSEA                                          0.043       0.030
  90 Percent confidence interval - lower         0.000       0.000
  90 Percent confidence interval - upper         0.155       0.149
```

```
  P-value H_0: RMSEA <= 0.050                    0.482       0.524
  P-value H_0: RMSEA >= 0.080                    0.372       0.332

  Robust RMSEA                                               0.031
  90 Percent confidence interval - lower                     0.000
  90 Percent confidence interval - upper                     0.153
  P-value H_0: Robust RMSEA <= 0.050                         0.519
  P-value H_0: Robust RMSEA >= 0.080                         0.342

Standardized Root Mean Square Residual:

  SRMR                                           0.120       0.120

Parameter Estimates:

  Standard errors                             Robust.sem
  Information                                   Expected
  Information saturated (h1) model            Structured

Latent Variables:
                        Estimate  Std.Err  z-value  P(>|z|)   Std.lv  Std.all
  vascular_density =~
    L1_ParaFove_pr         1.000                                4.647    1.000
  executive_function =~
    updating               1.000                                1.000    1.000
  updating =~
    Nback.RT_CV            1.000                                4.409    0.790
    nback.ACC_wm           1.608    0.543    2.963    0.003    7.091    0.458
    Nback.menRT_wm       -32.752    5.638   -5.809    0.000 -144.407   -0.962

Regressions:
                       Estimate  Std.Err  z-value  P(>|z|)   Std.lv  Std.all
  executive_function ~
    vsclr_dnst (c)      -0.156    0.135   -1.156    0.247   -0.165   -0.165
  HK_percent_pre ~
    vsclr_dnst (a)       0.160    0.153    1.046    0.295    0.744    0.203
  executive_function ~
    HK_prcnt_p (b)       0.612    0.217    2.816    0.005    0.139    0.509

Covariances:
                        Estimate  Std.Err  z-value  P(>|z|)   Std.lv  Std.all
 .executive_function ~~
    Age                   -4.228    2.468   -1.713    0.087   -1.109   -0.313
    Education              1.290    1.857    0.695    0.487    0.338    0.106
  vascular_density ~~
    RRsys                -18.645    8.415   -2.216    0.027   -4.012   -0.262

Variances:
                   Estimate  Std.Err  z-value  P(>|z|)   Std.lv  Std.all
   .L1_ParaFove_pr    0.000                                0.000    0.000
   .Nback.RT_CV      11.676    4.939    2.364    0.018   11.676    0.375
   .nback.ACC_wm    189.671   56.221    3.374    0.001  189.671    0.790
   .Nback.menRT_wm 1658.423 3391.767    0.489    0.625 1658.423    0.074
   .HK_percent_pre   12.894    2.477    5.205    0.000   12.894    0.959
    Age              12.562    2.542    4.942    0.000   12.562    1.000
    Education        10.180    4.584    2.221    0.026   10.180    1.000
    RRsys           235.046   69.566    3.379    0.001  235.046    1.000
    vascular_dnsty   21.599    4.915    4.395    0.000    1.000    1.000
   .executiv_fnctn   14.538    5.126    2.836    0.005    0.748    0.748
   .updating          0.000                                0.000    0.000

Defined Parameters:
                   Estimate  Std.Err  z-value  P(>|z|)   Std.lv  Std.all
    ab                0.098    0.107    0.914    0.361    0.103    0.103
    total            -0.058    0.171   -0.341    0.733   -0.061   -0.061
```

```
# Model 2
Mediation <- '# Mediation approach for executive_function ~ vascular density with haematocrit as mediator
                 vascular_density =~ L1_ParaFovea_pre
              # executive_function as latent variable
                 executive_function =~ MMSE_overall_pre
              # co-variances
                 executive_function ~~ Age
                 executive_function ~~ Education
                 vascular_density ~~ RRsys
              # direct effect
                 executive_function ~ c*vascular_density
              # haematocrit as Mediator
                 HK_percent_pre ~ a*vascular_density
                 executive_function ~ b*HK_percent_pre
              # indirect effect (a*b)
                 ab := a*b
              # total effect
                 total := c + (a*b)';

# call
model_fit <- sem(Mediation, data=mydata, estimator="mlm")
summary(model_fit, fit.measures = TRUE, standardized=TRUE)

lavaan 0.6-19 ended normally after 56 iterations

  Estimator                                         ML
  Optimization method                           NLMINB
  Number of model parameters                        12

                                                  Used       Total
  Number of observations                            38          41

Model Test User Model:
                                              Standard      Scaled
  Test Statistic                                 9.558       8.612
  Degrees of freedom                                 9           9
  P-value (Chi-square)                           0.387       0.474
  Scaling correction factor                                  1.110
    Satorra-Bentler correction

Model Test Baseline Model:

  Test statistic                                16.381      16.313
  Degrees of freedom                                15          15
  P-value                                        0.357       0.362
  Scaling correction factor                                  1.004

User Model versus Baseline Model:

  Comparative Fit Index (CFI)                    0.596       1.000
  Tucker-Lewis Index (TLI)                       0.327       1.492

  Robust Comparative Fit Index (CFI)                         1.000
  Robust Tucker-Lewis Index (TLI)                            1.544

Loglikelihood and Information Criteria:

  Loglikelihood user model (H0)               -639.009    -639.009
  Loglikelihood unrestricted model (H1)       -634.230    -634.230

  Akaike (AIC)                                1302.018    1302.018
  Bayesian (BIC)                              1321.669    1321.669
  Sample-size adjusted Bayesian (SABIC)       1284.148    1284.148

Root Mean Square Error of Approximation:

  RMSEA                                          0.040       0.000
  90 Percent confidence interval - lower         0.000       0.000
  90 Percent confidence interval - upper         0.190       0.170
  P-value H_0: RMSEA <= 0.050                    0.463       0.558
```

```
  P-value H_0: RMSEA >= 0.080                    0.430       0.336

  Robust RMSEA                                               0.000
  90 Percent confidence interval - lower                     0.000
  90 Percent confidence interval - upper                     0.187
  P-value H_0: Robust RMSEA <= 0.050                         0.542
  P-value H_0: Robust RMSEA >= 0.080                         0.365

Standardized Root Mean Square Residual:

  SRMR                                           0.111       0.111

Parameter Estimates:

  Standard errors                             Robust.sem
  Information                                   Expected
  Information saturated (h1) model            Structured

Latent Variables:
                        Estimate  Std.Err  z-value  P(>|z|)   Std.lv  Std.all
  vascular_density =~
    L1_ParaFove_pr         1.000                                4.583    1.000
  executive_function =~
    MMSE_overll_pr         1.000                                1.355    1.000

Regressions:
                       Estimate  Std.Err  z-value  P(>|z|)   Std.lv  Std.all
  executive_function ~
    vsclr_dnst (c)        0.076    0.039    1.969    0.049    0.257    0.257
  HK_percent_pre ~
    vsclr_dnst (a)        0.115    0.152    0.755    0.450    0.526    0.140
  executive_function ~
    HK_prcnt_p (b)       -0.016    0.054   -0.295    0.768   -0.012   -0.044

Covariances:
                        Estimate  Std.Err  z-value  P(>|z|)   Std.lv  Std.all
 .executive_function ~~
    Age                   -0.144    0.768   -0.187    0.852   -0.110   -0.031
    Education              0.458    0.540    0.848    0.396    0.350    0.100
  vascular_density ~~
    RRsys                -20.768    8.151   -2.548    0.011   -4.532   -0.291

Variances:
                   Estimate  Std.Err  z-value  P(>|z|)   Std.lv  Std.all
   .L1_ParaFove_pr    0.000                                0.000    0.000
   .MMSE_overll_pr    0.000                                0.000    0.000
   .HK_percent_pre   13.867    2.437    5.689    0.000   13.867    0.980
    Age              12.147    2.371    5.124    0.000   12.147    1.000
    Education        12.186    4.780    2.549    0.011   12.186    1.000
    RRsys           242.922   66.830    3.635    0.000  242.922    1.000
    vascular_dnsty   21.003    4.676    4.492    0.000    1.000    1.000
   .executiv_fnctn    1.716    0.339    5.067    0.000    0.935    0.935

Defined Parameters:
                   Estimate  Std.Err  z-value  P(>|z|)   Std.lv  Std.all
    ab               -0.002    0.006   -0.300    0.764   -0.006   -0.006
    total             0.074    0.039    1.889    0.059    0.251    0.251
```

```
# Model 3
Mediation <- '# Mediation approach for executive_function ~ vascular density with haematocrit as mediator
              vascular_density =~ L1_ParaFovea_pre
        # executive_function as latent variable
             executive_function =~ inhibition + MMSE_overall_pre
             inhibition  =~ Simon.meanRT_inh
        # co-variances
             executive_function ~~ Age
             executive_function ~~ Education
             vascular_density ~~ RRsys
        # direct effect
              executive_function ~ c*vascular_density
        # haematocrit as Mediator
             HK_percent_pre ~ a*vascular_density
             executive_function ~ b*HK_percent_pre
        # indirect effect (a*b)
             ab := a*b
        # total effect
              total := c + (a*b)';
# call
model_fit <- sem(Mediation, data=mydata, estimator="mlm")
summary(model_fit, fit.measures = TRUE, standardized=TRUE)

lavaan 0.6-19 ended normally after 270 iterations

  Estimator                                         ML
  Optimization method                           NLMINB
  Number of model parameters                        15

                                                  Used       Total
  Number of observations                            38          41

Model Test User Model:
                                              Standard      Scaled
  Test Statistic                                12.408      10.858
  Degrees of freedom                                13          13
  P-value (Chi-square)                           0.495       0.623
  Scaling correction factor                                  1.143
    Satorra-Bentler correction

Model Test Baseline Model:

  Test statistic                                20.949      21.797
  Degrees of freedom                                21          21
  P-value                                        0.462       0.411
  Scaling correction factor                                  0.961

User Model versus Baseline Model:

  Comparative Fit Index (CFI)                    1.000       1.000
  Tucker-Lewis Index (TLI)                     -17.760       5.339

  Robust Comparative Fit Index (CFI)                         1.000
  Robust Tucker-Lewis Index (TLI)                            6.159

Loglikelihood and Information Criteria:

  Loglikelihood user model (H0)               -825.416    -825.416
  Loglikelihood unrestricted model (H1)       -819.212    -819.212

  Akaike (AIC)                                1680.832    1680.832
  Bayesian (BIC)                              1705.396    1705.396
  Sample-size adjusted Bayesian (SABIC)       1658.495    1658.495

Root Mean Square Error of Approximation:

  RMSEA                                          0.000       0.000
  90 Percent confidence interval - lower         0.000       0.000
  90 Percent confidence interval - upper         0.155       0.129
  P-value H_0: RMSEA <= 0.050                    0.584       0.720
```

```
  P-value H_0: RMSEA >= 0.080                    0.299       0.181

  Robust RMSEA                                               0.000
  90 Percent confidence interval - lower                     0.000
  90 Percent confidence interval - upper                     0.147
  P-value H_0: Robust RMSEA <= 0.050                         0.693
  P-value H_0: Robust RMSEA >= 0.080                         0.219

Standardized Root Mean Square Residual:

  SRMR                                           0.105       0.105

Parameter Estimates:

  Standard errors                             Robust.sem
  Information                                   Expected
  Information saturated (h1) model            Structured

Latent Variables:
                        Estimate  Std.Err  z-value  P(>|z|)   Std.lv  Std.all
  vascular_density =~
    L1_ParaFove_pr         1.000                               4.583    1.000
  executive_function =~
    inhibition             1.000                               0.753    0.753
    MMSE_overll_pr        -0.014    0.017   -0.801    0.423   -0.349   -0.258
  inhibition =~
    Simon.menRT_nh         1.000                              33.871    1.000

Regressions:
                       Estimate  Std.Err  z-value  P(>|z|)   Std.lv  Std.all
  executive_function ~
    vsclr_dnst (c)       -0.887    0.979   -0.906    0.365   -0.159   -0.159
  HK_percent_pre ~
    vsclr_dnst (a)        0.115    0.152    0.755    0.450    0.526    0.140
  executive_function ~
    HK_prcnt_p (b)        1.702    0.942    1.807    0.071    0.067    0.251

Covariances:
                        Estimate  Std.Err  z-value  P(>|z|)   Std.lv  Std.all
 .executive_function ~~
    Age                  -22.591   17.992   -1.256    0.209   -0.922   -0.265
    Education            -16.685   13.757   -1.213    0.225   -0.681   -0.195
  vascular_density ~~
    RRsys                -20.768    8.151   -2.548    0.011   -4.532   -0.291

Variances:
                   Estimate  Std.Err  z-value  P(>|z|)   Std.lv  Std.all
   .L1_ParaFove_pr    0.000                               0.000    0.000
   .MMSE_overll_pr    1.706    0.368    4.635    0.000    1.706    0.933
   .Simon.menRT_nh    0.000                               0.000    0.000
   .HK_percent_pre   13.867    2.437    5.689    0.000   13.867    0.980
    Age              12.149    2.371    5.125    0.000   12.149    1.000
    Education        12.188    4.780    2.550    0.011   12.188    1.000
    RRsys           242.923   66.830    3.635    0.000  242.923    1.000
    vascular_dnsty   21.003    4.676    4.492    0.000    1.000    1.000
   .executiv_fnctn  600.403  739.033    0.812    0.417    0.923    0.923
   .inhibition      496.602  824.600    0.602    0.547    0.433    0.433

Defined Parameters:
                   Estimate  Std.Err  z-value  P(>|z|)   Std.lv  Std.all
    ab                0.195    0.242    0.806    0.420    0.035    0.035
    total            -0.692    0.934   -0.741    0.459   -0.124   -0.124
```
